# Steroid hormone levels and bone mineral density in women over 65 years of age

**DOI:** 10.1038/s41598-023-32100-x

**Published:** 2023-03-25

**Authors:** Elsa Nunes, Eugenia Gallardo, Sara Morgado-Nunes, José Fonseca-Moutinho

**Affiliations:** 1 Centro de Investigação em Ciências da Saúde, Universidade da Beira Interior, Avenida Infante D. Henrique, 6200-506 Covilhã, Portugal; 2 Laboratório de Fármaco-Toxicologia, UBIMedical, Universidade da Beira Interior, 6200-284 Covilhã, Portugal; 3 Escola Superior de Gestão, Instituto Politécnico de Castelo Branco, Avenida Pedro Álvares Cabral 12, 6000-084 Castelo Branco, Portugal

**Keywords:** Steroid hormones, Osteoporosis

## Abstract

Previous studies using immunoassays for steroid measurements have focused on the association between steroid hormone levels and bone mineral density (BMD) in postmenopausal women, obtaining contradictory results. This study aimed to assess this association using a highly sensitive bioanalytical method. A total of 68 postmenopausal women, aged 65–89 years, were enrolled in a cross-sectional study. Measurements of the BMD of the hip and lumbar spine were performed using dual energy X-ray absorptiometry, and serum hormone levels were quantified by gas chromatography and tandem mass spectrometry. Associations between estradiol (E2), testosterone, dehydroepiandrosterone (DHEA), androstenedione and T score levels of the hip and lumbar spine were evaluated, after adjustment for confounding variables. The analysis revealed a statistically significant association between testosterone and the T score of the hip (*p* = 0.035), but not that of the lumbar spine. No statistically significant associations were found between E2, DHEA, androstenedione and the T scores of the hip and the lumbar spine. Using a highly sensitive hormone assay method, our study identified a significant association between testosterone and BMD of the hip in women over 65 years of age, suggesting that lower testosterone increases the risk of osteoporosis.

## Introduction

As average life expectancy increases, more women will suffer the consequences of bone loss and osteoporosis. In recent decades, rather than being seen as an unavoidable disease in older women, osteoporosis has come to be seen as a preventable disease, and much progress has been made in its treatment. Osteoporosis is estimated to affect one-fifth of women aged 70, two-fifths of women aged 80, and three-fifths of women aged 90^1^. A recent meta-analysis reported that the worldwide prevalence of osteoporosis in the elderly women is 35.3%^2^.

Since Albright’s pioneering studies^3^, the association between estrogen deficiency and osteoporosis has well been recognized, and for some time the theory of estrogen-centered pathogenesis of postmenopausal osteoporosis prevailed^4^. In the past decade a shift in paradigm was observed, and new evidence revealed a possible role for androgens in the prevention of osteoporosis.

Androgens affect bone directly via interactions with androgen receptors, and indirectly via binding to estrogen receptors α and β after aromatization in fat or other tissues^5^. Furthermore, in postmenopausal women the combined treatment of androgens plus estrogens revealed more efficacy in increasing bone mineral density (BMD) than isolated estrogen^6,7^.

Menopause is associated with a 70% decline in adrenal androgens, including dehydroepiandrosterone (DHEA), which is converted at various levels into active androgens and/or estrogens in specific peripheral tissues by the process of intracrinology^8^. For decades, there has been controversy over whether the postmenopausal ovary is an androgen production site^9–12^. Postmenopausal bilateral oophorectomy was related to lower levels of BMD^13^ and to an increased risk of osteoporotic fractures^14^. More recently, it was stated that around 20% of serum DHEA originates from the postmenopausal ovary^15^, which in accordance with the intracrinology theory could explain the impact of postmenopausal oophorectomy on androgen levels. However, studies that have examined the association of androgens and bone mineral density have shown contradictory results, and the low specificity of the immunoassays used may have contributed. Although most of the studies revealed an association between androgens, particularly testosterone and BMD, others have failed to reach this conclusion.

In the 1960s, 70s and 80s, radioimmunoassay (RIA) was the main technique used for the dosing of steroid hormones. Despite the high throughput presented by these methods, the use of radioisotopes makes decontamination mandatory. Currently, non-radioactively labeled detection techniques (such as chemiluminescence or electrochemiluminescence) are widely implemented. Instruments for immunoassay-based methods are relatively easy to use, while sample preparation steps are not required and the cost is reasonable. However, these assays lack specificity due to the cross-reactivity of the antibodies with other steroid hormones^16^.

Mass spectrometry based (MS-based) techniques are now the gold standard for measuring steroid hormones in postmenopausal women, as they have greater accuracy and specificity than immunoassays, due to the very low serum concentrations of these hormones in the postmenopausal period^17^. Although high performance liquid chromatography and tandem mass spectrometry (LC–MS/MS) has become the preferred method for simple bioanalysis of an extended range of compound classes, gas chromatography and tandem mass spectrometry (GC–MS/MS) has higher accuracy, precision, sensitivity and specificity when it comes to measuring estrogen and androgens in the postmenopausal period^18^.

The goal of this study is to determine if lower levels of testosterone, androstenedione and DHEA are in fact associated with lower levels of bone mineral density in older women, using GC–MS/MS, a highly sensitive bioanalytical assay for steroid measurements in the postmenopausal period.

## Materials and methods

A cross-sectional study was conducted after approval by the Ethics Committee of Hospital Amato Lusitano, a Portuguese tertiary hospital. This study was carried out in accordance with relevant local regulations and the Declaration of Helsinki. Written informed consent was obtained for each participant. Between 2017 and 2019, 68 women over 65 years old evaluated in a gynecology consultation met the criteria for the performance of bone densitometry, according to the general health department`s guidelines. In Portugal, there is a government norm that recommends that all women over 65 should undergo bone densitometry of the lumbar spine and femoral neck using DXA technology (dual energy X-ray absorptiometry). For all patients included in this study, measurement of the bone mineral density (BMD) of the lumbar spine and femoral neck was performed in the same center using a GE Lunar Prodigy DXA system (GE Healthcare, Madison, WI, USA).

The inclusion criteria were women over 65 years of age who had intact ovaries at the time of menopause. Adnexal pathology was excluded by performing transvaginal ultrasound to all the participants. Current or past users of systemic hormonal therapy or corticosteroid treatment were excluded. Other exclusion criteria included history of taking any medication for osteoporosis, chronic hepatic or renal diseases, and history of endocrine or rheumatologic diseases.

One blood sample was obtained from each woman between 8 and 10 am. All blood samples were centrifuged within 1 h of collection to separate serum, which was stored at − 80 °C and protected from light until analysis. The studied compounds, dehydroepiandrosterone (DHEA), androstenedione, 17β-estradiol (E2), and testosterone were quantified by solid phase extraction (SPE) and gas chromatography and tandem mass spectrometry (GC–MS/MS). Briefly, 1 mL of plasma was diluted with 1 mL of phosphate buffer saline (PBS) (pH = 7) and spiked with 100 µL of internal standard (DHEA-d6). SPE cartridges (Oasis® HLB 3 cc, Waters, USA) were conditioned with 2 mL of methanol and 2 mL of 0.1% acetic acid. After passing of the sample through the cartridge, this was washed with 2 mL of deionized water. The columns were then dried under full vacuum for 30 min, and the analytes were eluted with 2 mL methanol. The extracts were concentrated to dryness under a gentle nitrogen stream; then, they were dissolved in 20 μL of methanol, from which a 3 μL aliquot was injected into the GC–MS/MS system. The remaining residue was further evaporated to dryness under a gentle nitrogen stream at 36 °C, and 20 μL of *N*,*O*-Bis(trimethylsilyl)trifluoroacetamide (BSTFA) was added. Derivatization took place in a domestic digital microwave oven (Candy CMG 2017 M, Portugal) for 2 min at 800 W, and 3 μL was injected. This step was deemed necessary because some of the analytes under study (E2 and T) present active moieties and need to be derivatized before analysis by GC-based procedures.

The statistical analysis software used was SPSS 27.0. Descriptive statistics were reported as means ± standard deviation (SD) for continuous variables and as frequencies (%) for categorical variables. Statistical analyses were obtained using Pearson`s correlations to examine associations between variables. In order to analyze the joint effect of the variables under study in the T score of the hip and in the T score of the lumbar spine, two multiple linear regression analyses were performed: one with T score of the hip as the dependent variable and the other with T score of the lumbar spine as the dependent variable. The independent variables were “Age”, “Race”, “Body mass index” (Kg/m2), “Regular alcohol habits”, “Smoking habits”, “Age of menarche”, “Type of menstrual cycles”(regular vs irregular), “Parity”, “Age of menopause”, “Years since menopause”, “Vaginal estrogen use”, “Estradiol levels (ng/mL)”, “Testosterone levels (ng/mL)”, “DHEA levels (ng/mL)”, and “Androstenedione levels (ng/mL)”. Qualitative variables were treated as dummy variables, and the regression method used was the stepwise, in which only regression variables significantly related to the dependent variable entered the regression model. These variables were entered successively according to their degree of association with the dependent variable. A *p* value of 0.05 or less was considered statistically significant.

## Results

Table 1 presents the demographic and laboratorial parameters of the participants. The majority of patients were Caucasian (98.5%). Obesity was confirmed in 44.1% of patients: 26.5% obesity class I, 13.2% obesity class II, 4.4% obesity class III; 41.2% of the patients were overweight, and only 14.7% had a normal weight. Most patients (98.5%) did not have alcoholic or smoking habits. Regular menstrual cycles throughout their reproductive life were reported by 88.2% of women. Only 7.4% of women were nulliparous and 75% had given birth to 2 or more children. The mean age of menopause was 50.2 years. The number of years since menopause (years from menopause to date of blood collection) was on average 22 years and in 40 women (58.8%) it was over 20 years. Vaginal estrogen cream was used two to three times weekly by 37 women (54.4%).

**Table 1:** Demographic characteristics and mean hormone levels of the participants (n = 68).

	Frequency n (%)	Mean	SD	Minimum	Maximum
Age (years)		72.2	6.6	65	89
Race (Caucasian)	67 (98.5%)				
BMI (kg/m^2^)		29.7	5.2	20	45
Underweight (< 18.5 kg/m^2^)	0 (0%)				
Normal weight (18.5–24.9 kg/m^2^)	10 (14.7%)				
Overweight (25–29.9 kg/m^2^)	28 (41.2%)				
Obesity class I (30–34.9 kg/m^2^)	18 (26.5%)				
Obesity class II (35–39,9 kg/m^2^)	9 (13.2%)				
Obesity class III (**≥ **40 kg/m^2^)	3 (4.4%)				
Age of menarche (years)		12.9	1.6	10	16
Menstrual cycles					
Regular	60 (88.2%)				
Irregular	8 (11.8%)				
Parity		1.9	0.9	0	4
Nulliparous	5 (7.4%)				
Multiparous					
1	12 (17.6%)				
2	34 (50%)				
> 2	17 (25%)				
Age of menopause (years)		50.2	4.4	37	58
Years since menopause		22.0	7.3	10	38
10–19	28 (41.2%)				
20–29	29 (42.6%)				
** ≥ **30	11 (16.2%)				
Regular alcohol habits	1 (1.5%)				
Smoking habits	1 (1.5%)				
Vaginal estrogen use	37 (54.4%)				
E2 (ng/mL)		0.94	2.49	0.05	18.00
Testosterone (ng/mL)		1.66	3.11	0.50	17.19
DHEA (ng/mL)		9.89	5.01	1.82	32.24
Androstenedione (ng/mL)		1.31	1.02	0.10	5.55

Controlling for all possible confounding variables (age, race, BMI, alcohol and smoking habits, age of menarche, type of menstrual cycles, parity, age of menopause, years since menopause, and vaginal estrogen use), positive correlations were found between the T score of the lumbar spine and the femoral neck and all four tested steroid hormones (Table 2). However, only the positive correlation between the testosterone concentration and the T score of the hip was statistically significant (*p* = 0.035).

**Table 2:** Pearson`s correlation coefficients for hormone levels and T score hip and lumbar spine.

Hormone levels	T score hip		T score lumbar spine	
	R	P	R	P
E2	0.162	0.234	0.144	0.289
Testosterone	0.283	0.035	0.155	0.254
DHEA	0.151	0.266	0.04	0.769
Androstenedione	0.245	0.069	0.131	0.337

Variables controlled: age, race, BMI, alcohol, smoking habits, age of menarche, type of menstrual cycles, parity, age of menopause, years since menopause, vaginal estrogen use.

By multiple linear regression analysis, it was found that testosterone and BMI positively affected the T score of the hip. Age negatively affected the T score of the hip, being the most predictive variable (Table 3). The type of regression used was stepwise and therefore only the statistically significant variables were expressed in the regression models. The first variable to enter the model was Age (Model 1), followed by Testosterone (Model 2) and BMI (Model 3). As the variables entered the models, the coefficients remained approximately constant, which means that there are no strong dependency relationships between these variables and therefore multicollinearity problems were not detected. The ANOVA table shows that, globally, the adjusted models are statistically significant and the proportion of variance explained by the regression models increased as successive variables entered the models (Table 4). We have also observed that only BMI positively affected the T score of the lumbar spine (Table 5) and the model is statistically significant (Table 6).

**Table 3:** Beta coefficients for testosterone, age and BMI in adjusted models of prediction of T score of the hip, using stepwise regression.

Model		β	Std. error	*P*	95% CI
1	Age	− 0.056	0.020	0.007	− 0.097; − 0.016
2	Age	− 0.051	0.020	0.012	− 0.091; − 0.011
	Testosterone (ng/mL)	0.086	0.041	0.037	0.005; 0.167
3	Age	− 0.045	0.019	0.024	− 0.084; − 0.006
	Testosterone (ng/mL)	0.089	0.039	0.028	0.010; 0.167
	Body mass index (kg/m^2^)	0.053	0.024	0.029	0.006; 0.101

a. Dependent variable: T score hip.

b. Predictors: E2, testosterone, DHEA, androstenedione, age, race, BMI, alcohol, smoking habits, age of menarche, type of menstrual cycles, parity, age of menopause, years since menopause, vaginal estrogen use.

**Table 4:** ANOVA table for stepwise regression models adjusted for the dependent variable T score of the hip.

Model		Sum of squares	Df	Mean square	F	Sig
1	Regression	8.524	1	8.524	7.714	0.007b
	Residual	71.831	65	1.105		
	Total	80.355	66			
2	Regression	13.283	2	6.641	6.337	0.003c
	Residual	67.072	64	1.048		
	Total	80.355	66			
3	Regression	18.187	3	6.062	6.144	0.001d
	Residual	62.167	63	0.987		
	Total	80.355	66			

a. Dependent variable: T score hip.

b. Predictors: Age.

c. Predictors: Age, Testosterone levels (ng/mL).

d. Predictors: Age, Testosterone levels (ng/mL), Body mass index (Kg/m2).

**Table 5:** Beta coefficients for testosterone, age and BMI in adjusted models of prediction of T score of the lumbar spine, using stepwise regression.

Model		β	Std. error	*p*	95% CI
1	Body mass index (kg/m^2^)	0.124	0.038	0.002	0.049; 0.198

a. Dependent variable: T score lumbar spine.

b. Predictors: E2, testosterone, DHEA, androstenedione, age, race, BMI, alcohol, smoking habits, age of menarche, type of menstrual cycles, parity, age of menopause, years since menopause, vaginal estrogen use.

**Table 6:** ANOVA table for stepwise regression model adjusted for the dependent variable T score of the lumbar spine.

Model		Sum of squares	Df	Mean square	F	Sig
1	Regression	26.954	1	26.954	10.847	0.002b
	Residual	161.518	65	2.485		
	Total	188.472	66			

a. Dependent Variable: T score lumbar spine.

b. Predictors: Body mass index (kg/m^2^).

## Discussion

The main objective of this study was to assess the positive associations between serum E2, DHEA and androstenedione and the bone mineral density of the hip and lumbar spine, by using a more sensitive laboratorial technique than those used in previous studies. Our results showed a statistically significant association between testosterone and bone mineral density of the hip in women over 65 years. Although positive correlations were found between E2, DHEA and androstenedione and bone mineral density of the hip and lumbar spine, we did not find statistical significance after adjustments for possible confounding factors.

Older studies using immunoassays for steroid measurements are conflicting. Our results are in line with the studies that showed an association between testosterone and BMD of the hip^19–22^. Tok et al. in a sample of 147 postmenopausal women (mean age 52 years) found that serum free testosterone levels were correlated positively with the BMD at the lumbar spine and femoral neck^20^. Likewise, Van Geel et al. found the same associations in 329 postmenopausal women, after adjustment for age^22^.

Lambrinoudaki et al. in the study with the largest sample to date (884 postmenopausal women not on hormone therapy), found testosterone and androstenedione were significantly associated with BMD at the hip. This study also confirmed an association between estradiol and BMD of the lumbar spine and hip^21^. It should be noted that the mean age of the participants in this study was 52.4 years.

However, other previous studies have failed to demonstrate that association^23–26^. Murphy et al. found significant positive correlations between the free estradiol and testosterone indices and bone mineral density at all sites but these relationships remained significant only for the free estradiol index after adjustment for age and body mass index^23^. Greendale et al. in a large population-based study (The Rancho Bernardo Study) of elderly women (mean age of 72.2 years), found the association between bioavailable testosterone and BMD was statistically significant only at the ultradistal radius, after accounting for covariates (age, BMI, alcohol, thyroid hormone, thyazides, exercise, cigarette use, and estrogen use)^24^. The other study that included women over 65 as participants (223 women) found that free testosterone was positively related to hip BMD, but after excluding estrogen users the sample was reduced, and there was a decrease in the magnitude and statistical significance of that relationship, which was even more attenuated after adjusting for estradiol^25^.

Concerns about the specificity of immunoassays when serum steroid levels are low have led to implementation of MS-based techniques as the gold standard methodology for steroid hormone analysis. Mass spectrometry offers a unique identification profile of each of the study analytes, eliminating interferences, thus allowing greater sensitivity and specificity ^27^. GC–MS/MS has been reported to be the more precise and accurate than LC–MS/MS in this type of analysis^18^. Derivatization is necessary for some of these compounds when gas chromatographic methods are used, since it improves the sensitivity and resolution of the separation. This is necessary to achieve the low concentrations usually found in biological specimens^16,18^. Concerning LC-based procedures, ion suppression can be directly related to inadequate sample preparation, and it is a major problem of LC–MS/MS techniques^28^. For instance, concerning the determination of testosterone in plasma from postmenopausal women, Thakur et al. have stated that GC/MS–MS provides excellent sensitivity and specificity when compared to liquid chromatographic methods, and helps elucidating the pharmacokinetic parameters of testosterone-related therapy, allowing as well monitoring endogenous testosterone as a pharmacodynamic biomarker^18^. In fact there are different published works about the determination of these compounds using GC–MS/MS^29–32^. In this work excellent limits of detection and quantitation were achieved (0.05 ng/mL for E2; 0.1 ng/mL for A and DHEA; 0.5 ng/mL for T) using only 1 mL of sample. Our study is the first to analyze associations between sex steroid levels and bone mineral density using GC–MS/MS, and for that reason, a small number of patients were included, aiming to be a pilot study.

We did not find a statistical significance between estradiol and BMD, and this can be explained by the small sample size of women included in our study but also by the pathophysiology of osteoporosis. An earlier classification of osteoporosis, although no longer used, divided osteoporosis in 2 types. In type I osteoporosis, there was a more pronounced effect of estrogen deprivation. In older women with type II osteoporosis, other factors could be additionally responsible for bone loss^33^. Using immunoassays for steroid measurements, Slemenda et al. also found that in older postmenopausal women, depending on skeletal site, both higher testosterone and estrogen concentrations were associated with slower bone loss^19^ and Stone et al. demonstrated in 9704 community-dwelling white women over 65 years of age that estradiol levels > 10 pg/mL were associated with 0.1% annual hip bone loss and levels below 5 pg/mL with an average of 0.8%^34^. Today it is known that estrogen decline in menopause is predominantly associated with trabecular bone loss. In women over the age of 65, most bone loss is cortical, not trabecular^35^. This could explain the absence of association of E2 and BMD in our sample of older women.

We found a weak positive correlation, with no statistical significance, between DHEA and BMD of the hip and lumbar spine. In theory, being a prohormone for the synthesis of estradiol and androgens, DHEA should correlate positively to BMD. Recently, Jankowsi et al. concluded, in a pooled analysis of four clinical trials, that women on treatment with oral DHEA had increased lumbar spine and trochanter BMD and maintained total hip BMD^36^. However the relationship between endogenous DHEA and BMD is not well recognized. Most of the studies analyzed DHEA sulfate (DHEAS), and the results are discrepant, either showing a positive association with BMD^20,37,38^ or no association^21,23,39^.

The finding of a significant positive influence of testosterone in bone mineral density of the hip in older women should encourage further research into testosterone deficiency in elderly women, with a potential impact in the prevention and treatment of postmenopausal osteoporosis. The effects of testosterone on the bone of older postmenopausal women are not very well documented but it is known that testosterone may have direct effects on bone via the androgen receptor, or indirect effects via aromatization^5^. The prevalence and burden of hip fractures has increased with increasing average life expectancy, and is one of the most serious health care problems affecting older women. In Portugal, as in Europe, the lifetime probability of hip fracture in women aged 70 is around 15%^40^. In women above 65 years of age, hip fracture is responsible for a twofold increased mortality in the first year after its occurrence^41^.

The major limitations of this study are the small sample size and the cross-sectional design of the study, which does not allow us to make causal inferences. Despite the limitations, our study is pioneering, because it is the first study analyzing associations between sex steroid hormone levels and bone mineral density using GC–MS/MS. We have shown that GC–MS/MS is a conveyable technique for future larger prospective studies conducted to provide accurate evidence that in older postmenopausal women androgen deficit plays an important role in bone loss and senile osteoporosis.

## Data Availability

The datasets generated during and/or analyzed during the current study are available from the corresponding author on reasonable request.
